# The Role of the Endocrine System in the Regulation of Acid–Base Balance by the Kidney and the Progression of Chronic Kidney Disease

**DOI:** 10.3390/ijms25042420

**Published:** 2024-02-19

**Authors:** Glenn T. Nagami, Jeffrey A. Kraut

**Affiliations:** 1 Nephrology Section, VA Greater Los Angeles Healthcare System, Los Angeles, CA 90073, USA; jkraut@g.ucla.edu; 2 Department of Medicine, David Geffen School of Medicine, University of California, Los Angeles, CA 90095, USA

**Keywords:** metabolic acidosis, aldosterone, angiotensin II, endothelin, parathyroid hormone, glucocorticoids, insulin, thyroid hormone, growth hormone

## Abstract

Systemic acid–base status is primarily determined by the interplay of net acid production (NEAP) arising from metabolism of ingested food stuffs, buffering of NEAP in tissues, generation of bicarbonate by the kidney, and capture of any bicarbonate filtered by the kidney. In chronic kidney disease (CKD), acid retention may occur when dietary acid production is not balanced by bicarbonate generation by the diseased kidney. Hormones including aldosterone, angiotensin II, endothelin, PTH, glucocorticoids, insulin, thyroid hormone, and growth hormone can affect acid–base balance in different ways. The levels of some hormones such as aldosterone, angiotensin II and endothelin are increased with acid accumulation and contribute to an adaptive increase in renal acid excretion and bicarbonate generation. However, the persistent elevated levels of these hormones can damage the kidney and accelerate progression of CKD. Measures to slow the progression of CKD have included administration of medications which inhibit the production or action of deleterious hormones. However, since metabolic acidosis accompanying CKD stimulates the secretion of several of these hormones, treatment of CKD should also include administration of base to correct the metabolic acidosis.

## 1. Introduction

Hormones can affect acid–base balance by impacting processes that are involved in the production of acid, buffering of acid and excreting acid from the body. Approximately 1 mEq/kg body weight of net endogenous acid (NEAP) is produced in adults and 2–3 mEq/kg body weight in normal children each day. In addition, ~4500 mEq/day (180 L × 25 mEq/L) of bicarbonate is filtered by the glomerulus and reabsorbed by the renal tubules, predominately the proximal tubule. For each milliequivalent of acid that is produced by metabolism, an equal quantity of bicarbonate must be generated by the kidney to maintain the blood pH and serum bicarbonate concentration at normal levels of ~7.38 ± 0.02 and 25.4 ± 0.09 mEq/L in males and ~7.40 ± 0.02 and 24.4 ± 1.3 mEq/L in non-pregnant females, respectively.

Although changes in the external and internal pH of renal tubules are the major factors affecting acid–base regulation by the kidney, alterations in the concentrations of several hormones including aldosterone, angiotensin II, endothelin, parathyroid hormone, glucocorticoids, insulin, thyroid hormone, and growth hormone can also play important roles. These effects might be magnified, with damage to the kidney resulting in chronic kidney disease (CKD). Paradoxically, the adaptive changes in concentrations of hormones which augment renal acid secretion and bicarbonate reabsorption can have maladaptive effects on the kidney by accelerating the progression of CKD.

In this review, we summarize the regulation of renal acid–base balance in individuals with normal renal function and CKD with an emphasis on the role of the endocrine system on the various processes which determine overall acid–base balance. Although the changes in hormones augment the body’s response to an acid load in many cases, some hormones may contribute to the progression of CKD and/or CKD-associated disorders. Thus, targeting certain hormones could provide another option in the treatment of patients with CKD.

## 2. Regulation of Acid–Base Balance

### 2.1. Net Acid Production

**Figure 1:**
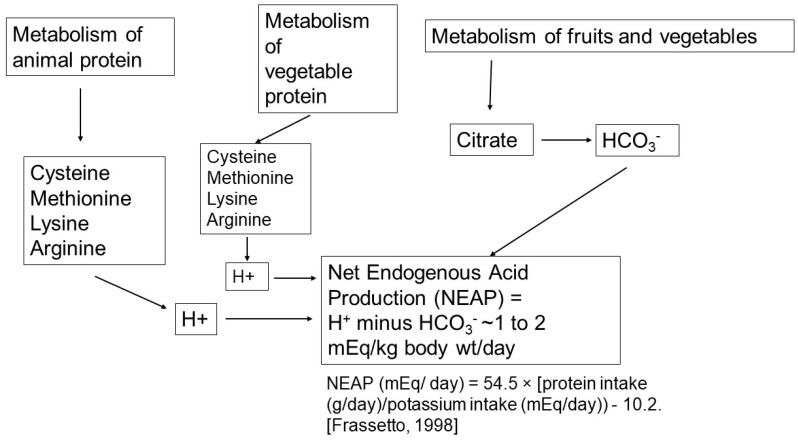
Net endogenous acid production (NEAP) is a composite of protons produced by metabolism of protein and base produced by metabolism of fruits and vegetables. H^+^ production is primarily due to metabolism of animal protein to amino acids cysteine, methionine, lysine and arginine. Smaller quantities of H^+^ are due to metabolism of vegetable protein of the same amino acids. Base is due to metabolism of fruits and vegetable primarily from citrate. Several formulae have been derived to estimate NEAP. A popular one is that proposed by Frassetto et al. [5].

Liver metabolism of ingested food results in H^+^ or HCO_3_^−^ production [1,2] (see Figure 1). On a typical North American diet, approximately 210 mEq of H^+^ is generated daily from the metabolism of the neutral sulfur-containing amino acids, methionine and cysteine to sulfate and H^+^ ions. In addition, the cationic amino acids, lysine, arginine, and some histidine residues are converted into neutral products and H^+^. Approximately 160 mEq/day of HCO_3_^−^ are generated from the metabolism of the amino acids, glutamate and aspartate, and organic anions such as citrate, gluconate, malate, acetate, and lactate. An additional 25–75 mEq of organic anions (potential base), half of which are metabolizable, are excreted in the urine. Therefore, NEAP each day is ~50 mEq. There is, however, often great variability among individuals with NEAP varying from 20 mEq to 120 mEq/day, primarily reflecting differences in diet content. When looking at dietary items which appear to separate people with a lower NEAP (<50 mEq/day) vs. those with high NEAP (>50 meq/day), there was a significantly higher consumption of vegetables and fruits in the group of individuals with lower NEAP [3]. In addition, some have suggested that differences in the individual microbiome (the bacterial population of the gastrointestinal tract) could affect the quantity of NEAP produced. The impact of dietary intake on acid–base balance in subjects with normal kidney function is exemplified by the studies of Kurtz et al. performed in normal subjects who were given different diets designed to produce net acid excretion between 14 and 154 mEq/day [4]. An inverse relationship between plasma [HCO_3_^−^] and endogenous acid load was found: the greater the acid load, the lower the plasma [HCO_3_^−^]. Frassetto et al. confirmed an impact of the level of dietary acid load on acid–base balance [5]. More recently in studies on African Americans who participated in the African American Study of Kidney Disease and Hypertension (AASK), higher NEAP was associated with lower serum bicarbonate concentrations. Moreover, for any given level of NEAP, the reduction in serum bicarbonate was greater with more severe reduction in kidney function (eGFR) [6].

In individuals with non-dialysis-dependent CKD, the generation of acid from diet does not appear to be affected by the presence of CKD. When individuals are maintained on a constant diet, net acid production rates appear to be equal in those with CKD and in those with normal kidney function [7]. Uribarri et al. [8,9,10] examined acid production rates in patients with non-dialysis CKD (GFR 19–33 m/min) or who were receiving chronic peritoneal dialysis or hemodialysis. In CAPD patients, in whom the plasma [HCO_3_^−^] was in the normal range, net H^+^ production was lower than that of individuals with normal renal function, but was similar to that of patients with CKD not receiving dialysis. The net H^+^ production rate in stable chronic hemodialysis patients with mild pre-dialysis hypobicarbonatemia (serum bicarbonate 21 to 23 meq/L) was reduced by ~50%. The reduction in net H^+^ production in patients receiving both renal replacement therapy modalities was attributed to a decrease in sulfuric acid generation from sulfur-containing amino acids of unclear cause and the retention of metabolizable organic anions, which were potential sources of base [9,10]. A fall in urinary excretion of metabolizable anions has also been described at earlier stages of CKD; whether this is due to the accompanying metabolic acidosis is not clear [11].

Dietary protein intake is often reduced in patients with CKD, which reduces NEAP. Also, the effect of changes in NEAP on acid–base parameters might be more profound in individuals with CKD than in those with normal renal function. For example, at entry into the Modification of Diet in Renal Disease (MDRD) study, serum [HCO_3_^−^] was inversely correlated with estimated protein intake (a major determinant of NEAP) such that there was a 1 mmol/L decease in bicarbonate for each gram per kilogram body weight increase in protein intake. Also, a 25% reduction in estimated dietary protein intake (from 1.01 to 0.74 g/kg body weight per day) in individuals with a mean GFR of 38 ± 9.2 mL/min per 1.73 m^2^ caused serum [HCO_3_^−^] to rise by approximately 1 mmol/L (0.91 ± 0.25 mmol/L). A similar effect of changes in dietary protein intake on serum [HCO_3_^−^] was detected in a study of African Americans with CKD [6]. In addition, in dialysis patients, a lower serum [HCO_3_^−^] was often observed in individuals ingesting the highest protein intake, whereas higher values of serum [HCO_3_^−^] are observed in those with low protein intake [12].

In summary, NEAP in individuals with CKD, both before and after initiation of maintenance dialysis, is usually normal or reduced. Thus, an elevated NEAP is rarely the major contributory factor in the development of hypobicarbonatemia. However, since NEAP is primarily correlated with dietary protein intake, any increase in protein intake can contribute to the development or worsening of metabolic acidosis and any reduction in protein intake below normal can lessen the severity of the metabolic acidosis. In addition, an increase in ingestion of fruits and vegetables, as sources of base, can reduce the net acid load for any level of protein intake. Indeed, increasing the intake of fruits and vegetables has been recommended as a means of providing base to patients with CKD not on dialysis [13].

### 2.2. Buffering of Acid by Tissues

The acid produced each day is first buffered by extracellular and cellular buffers in the muscles, bones, and kidneys. This results in a bicarbonate space, a measure of acid buffering, of approximately 50% body weight measured in kilograms. Muscle provides the largest reservoir of buffers as it accounts for 40% of the body weight and is rich in physiological intracellular buffers such as bicarbonate, phosphate, proteins and metabolic intermediates of glucose oxidation which by tissue buffering can mitigate the impact of acid loads on systemic pH in individuals with normal kidney function and CKD [14,15]. Interestingly, the severity of the acidity of the intracellular compartment observed in uremic individuals did not correlate tightly with the extracellular pH so that extracellular pH did not appear to be a good indicator of the pH within muscle cells (6.82 in intreated CKD compared to 7.04 in individuals without CKD) [14]. Bone can also serve to buffer acid [16]. With its large storage of potential base, bone could contribute to the maintenance of acid–base homeostasis in the setting of acid challenges [17]. Nevertheless, the quantitative contribution of bone to the maintenance of blood pH is not entirely clear [18]. When coupled with reduced kidney function, a reduced mass of buffering tissues (muscle and bone) which can occur with aging can result in a higher risk for acid retention and associated deleterious effects. As will be noted below, parathyroid hormone and thyroid hormone can modulate the degree of buffering by bone and muscle.

### 2.3. Bicarbonate Reabsorption and Generation by the Kidneys

The kidneys play a major role in maintaining acid–base balance. Depending on the quantity of animal protein ingested, metabolism generates approximately 1 mEq/kg body weight [4,15]. When NEAP is positive, it is imperative that the kidneys reclaim the large quantity of bicarbonate filtered by the glomeruli to prevent loss of base and produce new bicarbonate to compensate the NEAP from diet.

Although bicarbonate can be absorbed along the nephron, approximately 90% of the filtered bicarbonate is reabsorbed by the proximal tubule. The proximal tubule transports bicarbonate from lumen and back to blood through a series of biochemical and transport processes. Brush border membrane facing the lumen transports H^+^ from inside the cell to the lumen mostly via sodium–hydrogen exchange (predominately with the NHE3 isoform) and by a H^+^-ATPase proton pump. The H^+^ in the lumen reacts with HCO_3_^−^ to form H_2_CO_3_ which is converted to CO_2_ in the presence of a brush border carbonic anhydrase (CA IV). The CO_2_ passes into the cell via gas channels, where it encounters the intracellular carbonic anhydrase (CA II) and is converted back to HCO_3_^−^ and H^+^. The H^+^ is recycled back to the lumen, whereas the HCO_3_^−^ is transported back to the blood side of the proximal tubule via a basolateral Na^+^-HCO_3_^−^ co-transporter (NBCe1). As the transport of Na^+^ occurs against a steep electrochemical gradient (intracellular Na^+^ low and intracellular potential negative), the NBCe1-mediated transport occurs by transporting 3 anionic HCO_3_^−^ for each Na^+^ moved across the basolateral membrane [19]. Na^+^ is also transported by the ATP-driven Na^+^-K^+^ ATPase, thereby accomplishing net movement of Na^+^ and HCO_3_^−^ from lumen to peritubular capillaries. HCO_3_^−^ extrusion out the basolateral membrane can also occur via electroneutral basolateral HCO_3_^−^–chloride exchange [20], which could account for HCO_3_^−^ exit but not for additional Na+ efflux. In any case, defective bicarbonate reabsorption occurs with treatment with carbonic anhydrase inhibitors and in proximal RTA and in Fanconi’s syndrome.

The generation of new bicarbonate occurs as a result of the excretion of ammonium and titratable acid. Ammonia production and excretion are the main ways by which the kidney increases acid excretion in response to increased acid loads. Ammonia production is determined by delivery of substrate amino acids (normally glutamine) to the kidney, where they are converted in the proximal tubule to ammonium and through metabolism of the amino acid carbon skeleton to bicarbonate. Excretion of the ammonium into the final urine is important to prevent its return to the liver, where it can be detoxified to urea, a process that consumes bicarbonate. The production of titratable acid (i.e., H^+^ buffering to anions such as phosphate) is mainly driven by the delivery of phosphate to the distal nephron coupled with secretion of H^+^ in the collecting duct.

In chronic kidney disease (CKD), the ability of the kidney to compensate for the daily acid load may become impaired so that acid retention occurs. Although problems with bicarbonate reclamation can occur in some individuals with CKD [21], bicarbonate reabsorption is usually intact [22]. Therefore, the major reason for acid retention in CKD is a reduction in ammonia production and excretion [23,24]. In any case, when the acid production even slightly exceeds the net acid excretion rates, acid can accumulate in the interstitium and cells of the kidney and be buffered by bone so that reductions systemic blood bicarbonate and pH are not observed [25,26,27]. As the imbalance between acid production and acid excretion worsens, reductions in the serum bicarbonate and blood pH are seen.

The importance of avoiding acid accumulation is underscored by its many clinical consequences which include dysfunction of several organs (muscle [28,29], bone [18,30,31] and kidney [32]) and an increase in mortality [33]. We will now focus on the important role of several hormones in the response to acid challenges and also their potential contributory role to progressive kidney disease.

## 3. Roles of Hormones in Acid-Balance and in Ckd Progression

A variety of hormones including aldosterone, angiotensin II, endothelin, parathyroid hormone, glucocorticoids, insulin, antidiuretic hormone, thyroid hormone and growth hormone play roles in modulating these processes (see Table 1).

### 3.1. Aldosterone

Usually considered as a regulator of sodium and potassium balance, aldosterone is also a major modulator of acid excretion by the kidney. The role of aldosterone in the response of the body to acid loads is underscored by the effect of acute metabolic acidosis to increase circulating levels of aldosterone, thereby enhancing its effect on acid excretion by the kidney [34,35]. Aldosterone enhances sodium reabsorption in the collecting duct, thereby setting up a negative lumen potential that not only favors potassium secretion but also electrogenic H^+^ secretion via the vacuolar H^+^ ATPase located in the acid-secreting intercalated cells of the collecting duct. Aldosterone also has direct effects on translocation of H^+^-ATPases to the luminal membrane via a protein kinase C-dependent pathway [36,37] which increases the number of proton pumps. Furthermore, aldosterone enhances the luminal expression of the ammonia transporter RhCG in intercalated cells [38] which can interact with the vacuolar H^+^ ATPase to facilitate ammonia transport into the lumen [39]. Thus, cell-to-lumen flow of ammonia can be directly facilitated by the co-upregulation of the two transporters. On the other hand, reductions in aldosterone levels as seen in primary adrenal insufficiency [40] and hyporeninemic hypoaldosteronism [103] are associated with non-anion gap metabolic acidosis as well as hyperkalemia. The cause for reduced acid secretion in patients with reduced aldosterone levels and high potassium levels arise from the direct inhibition of kidney ammonia production by hyperkalemia. This concept is supported by studies showing that correction of the hyperkalemia can correct metabolic acidosis in such individuals [104,105].

In CKD, circulating aldosterone levels are generally elevated [41]. Elevated aldosterone levels in patients with CKD are associated with hemodynamic and profibrotic effects that lead to CKD progression regardless of the presence or absence of diabetes [42]. The increase in aldosterone levels appears to be due in part to accompanying acid retention, as both in humans and animals, provision of base reduces aldosterone levels and slows CKD progression [32,106].

Patients with diabetes and other conditions, however, may have low circulating aldosterone levels in the setting of hyporeninemic hypoaldosteronism [107]. It is important to recognize that many drugs that slow the progression of CKD such as angiotensin-converting enzyme inhibitors (ACEIs), angiotensin receptor blockers (ARBs) and mineralocorticoid receptor antagonists (MRAs) can cause hyperkalemia and hypobicarbonatemia. Therefore, careful monitoring of serum potassium and total CO_2_/bicarbonate levels as well as kidney function in patients receiving these agents is imperative.

### 3.2. Angiotensin II

Angiotensin II has direct effects on acid–base transporters and metabolism in the kidney. In the proximal tubule, angiotensin II, acting through its type 1 angiotensin receptor (AT1R), stimulates Na^+^-H^+^ exchange [44], Na^+^-HCO_3_^−^ cotransport [44,45] and ammonia production and secretion [46,108]. In the outer medullary collecting duct, angiotensin II acting through its AT1R increases vacuolar H^+^-ATPase activity in acid-secreting intercalated cells by enhancing trafficking of this transporter to the luminal membrane [47]. Furthermore, acid-loading promotes the effects of angiotensin II on the kidney by activation of the intrarenal renin–angiotensin system [43] and by increasing its effect on base generating processes. In mice given short-term acid loads, angiotensin II stimulated ammonia production and secretion by the proximal tubule to a higher degree than tubules obtained from non-acid loaded mice [109]. This enhanced effect was seen even though the unstimulated levels of ammonia production in short-term acid loaded mice were not perceptibly higher than the levels observed in control mice. It is unclear whether the enhancement of the effects of angiotensin II with acid loading is limited to the beneficial adaptive response to increase ammonia production and secretion or can extend to potentially nephropathic effects of angiotensin II on the progression of CKD.

In mice and humans with CKD, blocking AT1Rs is renoprotective, preventing progression of disease but also reducing the ability of the kidneys to fully enhance the excretion of ammonia in response to metabolic acidosis [40,109]. Increased intrarenal activation of the renin–angiotensin system in CKD has been associated with higher risk of progression [48], but could also represent a response to maintain the ability of the kidney to handle dietary acid loads in the face of reduced kidney function. In a study of risk factors for reduced serum bicarbonate in patients with CKD [110], the use of angiotensin-converting enzyme inhibitors or angiotensin receptor blockers was associated with a higher risk for having lower serum bicarbonate levels. As useful as they are in slowing the progression of CKD, ACEIs and ARBs may not prevent all of the adverse effects of acid retention so that serum bicarbonate/tCO_2_ should be monitored closely and low serum bicarbonate/tCO_2_ levels treated with base supplementation and/or with dietary adjustments to increase fruits and vegetables while reducing animal protein.

### 3.3. Endothelin

Endothelins are small peptide hormones, which are potent vasoconstrictors and are made by endothelial cells and other cells in the body [111]. The major and most-studied endothelin is endothelin-1 which in addition to its vasoconstrictor properties can have direct effects on the ability of the kidney to excrete acid by augmenting acid secretion by the proximal tubule [51,52] and collecting duct [49,53]. In the collecting duct, endothelin-1 may not only stimulate acid-secretion by type A intercalated caells but may also reduce secretion of bicarbonate in type b intercalated cells [50].

Endothelin-1 levels are increased with acid-retention and in CKD [55,56]. In CKD, acid-retention may also be the major driver for elevated endothelin levels. The increased endothelin-1 levels may serve to maintain adequate levels of acid excretion as kidney function declines, but endothelin-1 also has adverse proinflammatory and profibrotic effects, which may become maladaptive over time [56]. Increased endothelin levels lead to increased tubule-interstitial damage, inflammation, and fibrosis, which are associated with increased synthesis of fibronectin and collagen, effacement of the podocytes and a decline in GFR. As endothelin may be a factor contributing to progression of CKD, agents which block endothelin receptors are being used to slow the progression of CKD [54,59]. In addition, treatment of acid retention and metabolic acidosis can lower endothelin levels and reduce their harmful effect on kidney function [32].

### 3.4. Parathyroid Hormone

Parathyroid hormone (PTH) plays an important role in acid–base regulation both by making available bone carbonate buffer stores to counteract acid accumulation in CKD [17] and also by altering kidney transport processes involved in bicarbonate reclamation and net acid excretion [57,60]. PTH levels may be increased with metabolic and respiratory acidosis so that it may play an active role in acid–base homeostasis [61]. PTH has been associated with increased urinary bicarbonate losses possibly related to reduced proximal tubular bicarbonate reabsorption. This observation, however, was more likely due to increases in the filtered load of bicarbonate resulting from sustained elevations in serum bicarbonate levels associated with elevated PTH levels [60,112]. In response to acute metabolic acidosis, PTH levels increase with associated enhancement of phosphate excretion, titratable acid and ammonium excretion. The effect of acidosis to increase PTH secretion may be mediated through the reduction in the calcium-sensing receptor (CSR) activity by extracellular pH, thereby reducing its inhibitory effect on PTH secretion [58]. The mechanism by which extracellular pH alters CSR activity is unknown. Point mutations in candidate histidine residues in the CSR, which could theoretically convey pH sensitivity did not alter the CSR response to altered extracellular pH [58]. Parathyroidectomy markedly blunts baseline and acid-induced net acid excretion [62] as well as reducing cellular buffering [113].

Although animal studies suggested a positive correlation between parathyroid hormone and renal net acid excretion, a finding with strong biological plausibility, the evidence of such a tight relationship between PTH and phosphorus and titratable acid excretion in humans with CKD has been less clear. Studies involving the Chronic Renal Insufficiency Cohort (CRIC) showed that higher acid loads and metabolic acidosis were associated with increased serum phosphorus concentration and augmented phosphaturia, but not consistently associated with increased concentrations of PTH or FGF-23, two known phosphaturic hormones [63]. These results may have been confounded by variations in diet and kidney function. Also, direct measures of titratable acid excretion were not obtained in these studies. Rather, titratable acid excretion was estimated using the measured urine phosphorus concentration, and urine pH.

Epidemiologic studies have shown an association between the presence of secondary hyperparathyroidism and CKD progression and cardiovascular outcomes [64,114]. The causal relationship is unclear with higher PTH levels being more likely to occur with worsening kidney function [114]. The impact of secondary hyperparathyroidism on acid–base regulation in CKD may be related to its possible effect on bone buffering and on maintaining phosphate excretion, and thereby titratable acid excretion.

### 3.5. Glucocorticoids

Glucocorticoids play a key role in the body’s response to acid challenges. Yet, glucocorticoids have been shown to increase endogenous acid production, which could theoretically increase acid accumulation in the body if it were not for their effects to increase net acid excretion by the kidney [65]. In metabolic acidosis circulating glucocorticoid levels are elevated [66] which increase proximal tubular bicarbonate reabsorption and ammonia secretion which, in turn, prevent further falls in circulating bicarbonate levels. Glucocorticoids are necessary for optimal enhancement of Na^+^-H^+^ exchanger activity with metabolic acidosis [67], thereby ensuring optimal bicarbonate reclamation and ammonium secretion by the proximal tubule in this setting. Dexamethasone supplementation increases the apical membrane activity of the NHE-3 isoform of the Na^+^-H^+^ exchanger in brush border membranes [68]. Glucocorticoids are also important in enhancing the uptake and oxidation of glutamine, the major substrate for ammoniagenesis, by the proximal tubules [69]. The effects of glucocorticoids on distal acidification are less clear. In adrenalectomized rats, the provision of glucocorticoids increases urinary acidification, but this could be an indirect effect of an increased glomerular filtration rate causing increased delivery of phosphate and sodium to the distal nephron [115,116]. Studies on rabbit medullary collecting ducts after chronic treatment with glucocorticoid (dexamethasone) did not affect acid secretion by this segment, whereas mineralocorticoid did [117].

In addition to their effects on acid transport and ammonia secretion in the proximal tubule, with metabolic acidosis, glucocorticoids stimulate muscle proteolysis [118], providing amino acid substrates for increasing blood glutamine levels, thereby increasing delivery of glutamine to the kidney for ammoniagenesis [70]. These effects of glucocorticoids to foster the breakdown of muscle protein and contribute to sarcopenia could also eventually reduce tissue acid buffering capacity by reducing muscle mass.

In humans with CKD, the extraction of glutamine by the kidney is suppressed and its role as a substrate for ammoniagenesis is lessened [71]. The role of glucocorticoids in this shift of ammoniagenic substrates is unclear. Circulating glucocorticoid levels are increased in humans with CKD [72], and this may be related to reduced clearance of cortisol and shifts in peripheral metabolism. Higher rates of production may correlate with progression of CKD [119]. It is unknown whether resistance to the action of glucocorticoids in the kidney and proximal tubule may contribute to disturbed metabolism of glutamine. Resistance to glucocorticoids occurs in peripheral blood lymphocytes via a post-receptor mechanism [120]. It is unclear whether other tissues, including the kidney, display similar patterns of glucocorticoid resistance.

### 3.6. Insulin

Studies in animals and humans have demonstrated a role for insulin in the regulation of the transport of sodium by the kidney [121,122]. The effect on sodium excretion was not associated with changes in serum aldosterone levels [121]. Although it is known that metabolic acidosis and CKD can induce insulin resistance [73,74,75,122], insulin’s role in acid–base regulation under normal circumstances and in CKD is less clear. Administration of pharmacologic doses of insulin to dogs while maintaining constant serum glucose levels resulted in reduced distal acid excretion as estimated by a decline in the urine-to-blood pCO_2_ [123]. This occurred whether the serum potassium was maintained at a constant level or allowed to fall. In studies of patients with insulin-dependent diabetes, more physiological doses of insulin were used while maintaining euglycemia, and demonstrated a transient rise in urinary pH and bicarbonate excretion with a fall in ammonia and titratable acid excretion that was dose-dependent. Studies using the euglycemic clamp approach maintain serum glucose levels by increasing glucose infusion rates while insulin is maintained at a constant level. Therefore, some change in intracellular glucose flux would be anticipated, which could alter ammoniagenic processes in the kidney. In other studies, insulin had no significant effect on renal glutamine net balance, fractional excretion or release and uptake [124]. These findings suggested that changes in glutamine-driven renal ammonia production rates were unlikely to be affected by insulin. Taken together, the effects of insulin on acid excretion were likely due to modification of transport mechanisms which determine acid excretion rather than a direct effect on glutamine metabolism and renal ammoniagenesis.

In CKD, insulin resistance can contribute to the progression of CKD for several reasons. Insulin resistance with hyperinsulinemia can result in altered renal hemodynamics [75], sodium retention [77], altered renal albumin handling [78], mesangial cell growth [79] and renal fibrosis [76]. Acidosis in patients with CKD may contribute to insulin resistance and its correction can improve insulin resistance [125]. Further studies including a larger number of individuals with and without diabetes are needed to validate the role of insulin resistance in progression of CKD.

### 3.7. Antidiuretic Hormone

Evidence for a role of antidiuretic hormone (ADH) in acid–base regulation is limited. In patients with the syndrome of inappropriate ADH secretion the serum [HCO_3_^−^] is usually normal despite the potential diluting effects of water retention [88]. The maintenance of a normal serum [HCO_3_^−^] has been attributed to changes in aldosterone levels [89]. Nevertheless, ADH has been demonstrated to play a role in stimulating acid excretion by the kidney by increasing collecting duct H^+^ transport [60,126], which could theoretically augment bicarbonate generation by the kidney and boost serum [HCO_3_^−^]. In diabetic ketoacidosis, ADH levels may be initially elevated and fall with treatment [90]. It was, however, unclear whether the initially elevated levels contributed to the clinical or laboratory changes observed in these patients. It is unknown whether circulating concentrations of ADH or its co-secreted peptide copeptin are altered in other forms of metabolic acidosis in which the osmolality, hydration and volume status have remained normal.

ADH levels may be elevated in individuals with CKD with associated increases in serum osmolality as the azotemia of CKD progresses [91]. At the same time, the rise in ADH was associated with a reduction in urine cyclic AMP, tubular epithelial aquaporin 2 abundance and concentrating ability, all consistent with tubular resistance to the action of ADH.

The effect of ADH on the progression of chronic kidney disease has been demonstrated in patients with autosomal dominant polycystic kidney disease (ADPKD). The beneficial effect of blocking the V2 receptor of ADH with tolvaptan was most evident in patients with ADPKD who had enlarging kidney volume and rapidly progressive disease [92,127]. Interestingly, one observational study of 67 individuals treated with chronic tolvaptan for ADPKD showed an increase in serum bicarbonate levels with tolvaptan treatment [128]. The reason for an increase in bicarbonate concentration was unclear, but as net acid excretion was diminished in individuals treated with tolvaptan [81], the higher bicarbonate level did not result from enhanced acid excretion but rather from either increased base or reduced acid absorption into the body or reduced net endogenous acid production.

### 3.8. Thyroid Hormone

The presence of normal levels of thyroid hormone are important in allowing the kidney to respond optimally to acid challenges. In man, non-immune hypothyroidism is generally associated with a normal serum bicarbonate concentration, but when an acid load is given, a defect in distal nephron acidification can be observed [82]. Studies on rats have demonstrated that hypothyroidism was associated with reduced urinary acidification [83], which was likely due to a distal defect in acid secretion as estimated by a reduced urine-to-blood pCO_2_ gradient after bicarbonate loading [84]. Subsequent studies exploring the transport mechanisms of the renal acidification defect observed with thyroid hormone deficiency demonstrated that the acid–base transporters which appeared to be affected most were those located in the apical membrane of the proximal tubule. The sodium–hydrogen exchanger 3 (NHE3), the B2 subunit of the H^+^-ATPase and the sodium-phosphate co-transporter IIa (NaPi IIa) were reduced in hypothyroid rats, whereas the number of acid-secreting intercalated cells of the collecting duct was increase in a compensatory manner [80]. It should be pointed out that chronic metabolic acidosis can be associated with reduced thyroid hormone levels and elevate thyroid-stimulating hormone levels and these factors could further contribute to an altered response to acid loads [85].

In addition to the direct effects thyroid hormone has on acid–base transport processes in the kidney, reduced thyroid hormone levels may dampen the release of glutamine from muscles, which could reduce the flow of glutamine to kidneys and impair ammoniagenic response to an acid load [87]. Nevertheless, the effect of hypothyroidism on interorgan glutamine transport in metabolic acidosis or in CKD has not been directly examined.

CKD is associated with altered thyroid function [86]. There are low serum free tri-iodothyronine (T3) levels which have been attributed to altered peripheral conversion of T4 and which can be associated with elevated thyroid-stimulating hormone levels. Hypothyroidism can be associated with worse clinical outcomes (mortality, cardiovascular disease, physical function and body composition) [86]. The reduction in body muscle content could result in a reduced reservoir for amino acids to be delivered to the kidney for ammoniagenesis and reduced muscle tissue volume to serve as an acute buffer to acid loads. As patients with CKD have many reasons for reduced acid excretion, the independent effect of thyroid dysfunction on kidney acid–base handling in CKD is difficult to discern. In any case, correction of acidosis with base therapy improves thyroid axis indices [85,129].

### 3.9. Growth Hormone and Insulin-like Growth Factor 1

Growth hormone and insulin-like growth factor 1 (IGF-1) affect a variety of kidney functions including glomerular filtration rate and phosphate reabsorption [93] and can play a role in systemic acid–base balance [93,95]. In studies of rats, removal of the pituitary gland resulted in metabolic acidosis due to a decrease in net acid secretion, and treatment with growth hormone corrected the metabolic acidosis and restored renal acid excretion likely by improving renal tubular acid secretion [96] and ammoniagenesis [94]. Studies in canine isolated proximal tubules demonstrated a stimulatory effect of growth hormone on ammonia production [94]. The clinical relevance of the effect of low growth hormone levels and activity was observed in children with growth hormone deficiency and displayed mild metabolic acidosis which resolved with administration of growth hormone.

Human adults given an NH_4_Cl acid load were noted to have reduced IGF-1 levels due to a reduced hepatic stimulatory response to growth hormone while growth hormone levels were slightly higher with a greater rise in response to growth hormone stimulating factor than in non-acidotic controls [130]. When human subjects were given an NH_4_Cl acid load to induce metabolic acidosis and subsequently given a sustained period of growth hormone, ammonia excretion increased and serum bicarbonate level was partially corrected [95]. The latter study indicated that higher levels of growth hormone could increase IGF-1 levels and overcome the apparent resistance to the action of basal levels of growth hormone to stimulate ammonia production and acid excretion by the kidney. The mechanism of enhanced acid secretion was explored in subjects who were restricted in dietary sodium intake while being given an NH_4_Cl acid load and growth hormone [97]. Sodium restriction did not affect the stimulation of ammoniagenesis by growth hormone but did block urinary acidification and net acid excretion compared to subjects with higher sodium intakes. Distal sodium delivery to the collecting duct appeared to be important in stimulating sufficient proton secretion to promote urinary acidification and net acid excretion.

Growth hormone has been shown to exert physiological effects that are not mediated by IGF-1. The effect of growth hormone to stimulate ammoniagenesis in proximal tubule segments was demonstrated in the absence of IGF-1 in vitro [94], and the effect of growth hormone to stimulate collecting duct hydrogen ion secretion was not reproduced by the provision of IGF-1 [96]. Studies on rabbit proximal tubules showed no direct effect of either growth hormone or IGF-1 on volume and bicarbonate reabsorption, but IGF-1 did increase phosphate reabsorption [98]. As the somatic effects of growth hormone are mediated via IGF-1, disruption of the growth hormone/IFG-1 access could reduce muscle and bone mass, which could potentially affect the tissue buffering capacity in the body. The overall effect of growth hormone and IGF-1 on acid–base balance in the setting of metabolic acidosis would likely reflect the relative acid–base impact of acidosis on growth hormone and IGF-1 levels and their effects on acid–base transport and metabolic processes.

In severe CKD, although growth hormone levels may be normal or increased, there is resistance to growth hormone action due to reduced growth hormone receptor expression in tissues, post-receptor dysregulation, reduced IGF-1 levels and the presence of inhibitory proteins which inhibit the action of IGF-1 [99]. Whether CKD-induced resistance to growth hormone extends to its effect on acid–base functions of the kidney is unknown. Of note, although studies were carried out in experimental CKD [100], acromegalic individuals [101,102] and individuals with poorly controlled type 1 diabetes mellitus [131], high growth hormone levels can be associated with glomerular hyperfiltration, hypertrophy and glomerulosclerosis (the latter more rarely in humans), and chronic administration of growth hormone for the treatment of growth hormone deficiency has not been associated with progression of chronic kidney disease [132,133,134].

### 3.10. Effects of Acid pH Which May Contribute to CKD Progression in the Absence of Hormones

So far, the emphasis of this review has been to highlight the roles of various hormones in the regulation of acid–base balance and their potential impact on acid–base balance and CKD progression. However, there is evidence that changes in the level of acidity in the surrounding cellular environment can directly inflict kidney damage. In vitro models in which the extracellular milieu can be tightly controlled have used to examine the effects pH to induce potential inflammatory mediators. Lowering the pH of cultured RAW (macrophage-like) cells to 7 and 6.5 with hydrochloric acid resulted in an increased proinflammatory profile [135]. Such pH-induced changes could result in an inflammatory response, which could provoke further injury to neighboring kidney cells [136]. In cortical slices and isolated proximal tubules, exposure to pH 6.5 with low bicarbonate concentration resulted in a more oxidized nicotinamide adenine dinucleotide (NAD^+^) state and altered lipid metabolism, which resulted in tubular damage [137]. Although these studies have demonstrated provocative effects of low pH on potentially pathogenic factors in the kidney, the extreme reduction in pH applied in these studies would seem to lessen the applicability of the results to individuals that we see in our CKD clinics. As illustrated in this review, the design of in vivo studies on isolated acid–base status as a single causative factor would be challenging given the complex interplay among changes in acid–base status and various hormone levels.

## 4. Summary and Conclusions

**Figure 2:**
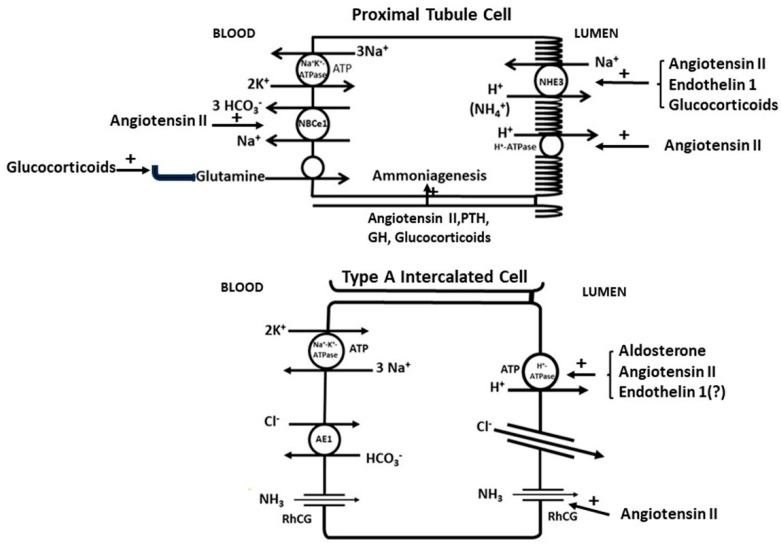
H^+^ retention and acidosis from different causes including CKD can result in increased levels of a number of hormones including aldosterone, angiotensin II, endothelin^_^1, parathyroid hormone (PTH), glucocorticoids and growth hormone (GH). Increased levels of each hormone can increase the ability of the body to handle acid loads through different mechanisms involving proximal tubule cells and type A intercalated collecting duct cells. Not shown are type B intercalated cells, which may be reduced in number and activity with acid loads.

Aldosterone, angiotensin II, endothelin, PTH and glucocorticoids play important roles in the regulation of acid–base balance by the kidney in patients with normal renal function. Other hormones such as insulin, growth hormone and thyroid hormone may also play a role under certain circumstances. In general, alterations in the activity of individual hormones can affect certain transporters and metabolic processes in the proximal and/or distal tubules to alter the kidney response to acidosis (see Figure 2). Their role in the regulation of acid–base balance in patients with CKD may become more important as renal function declines. Further, in some cases, development of metabolic acidosis can increase hormonal concentration or activity. In such cases, improvement in acid–base balance resulting from treatment with base is associated with a decrease in the hormonal concentrations.

**Figure 3:**
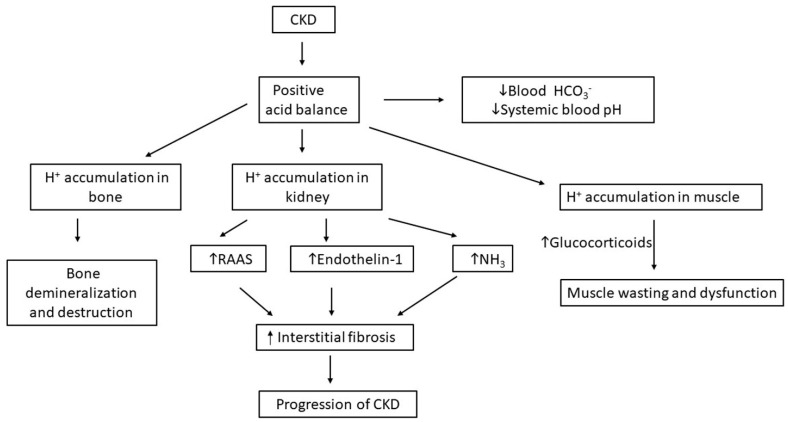
Effect of CKD on hormone secretion, renal acid excretion and progression of CKD. A complex interplay exists between development of CKD, hormone secretion, and progression of CKD. Increases in secretion of endothelin, aldosterone, and angiotensin II with CKD may also cause kidney damage. Glucocorticoids are also increased in CKD and acidosis and play a role in muscle catabolism; although they are initially shuttled to the kidney amino acids for ammoniagenesis, sarcopenia can result in reduced tissue buffering of acid. Treatment strategies that lessen these effects could slow progression of CKD. Direct effects of metabolic acidosis to cause kidney damage have been suggested by in vitro studies, but the extreme levels of low pH used in the studies reduced their applicability to most patients with CKD (RAAS: renin–angiotensin–aldosterone system).

A rise in the concentration of certain hormones can adversely affect kidney function and accelerate the progression of CKD (Figure 3). Administration of agents that block the action of such hormones or with base, which lowers the concentration of the hormones, can slow the progression of CKD. Therefore, administration of base to improve acid–base balance in patients with CKD and administration of agents that block the action of the hormones should be considered as an integral component of therapy.

## Figures and Tables

**Table 1:** Hormones Involved in Regulation of Acid–Base Balance and Their Impact on Progression of Chronic Kidney Disease.

Hormone	Effect of Acidosis on Serum Level or Action	Effect on Acid Base Balance	Role Acid–Base Balance in CKD	Effect on Progression of CKD
Aldosterone	Increased levels with metabolic acidosis [34,35]	Increases H^+^ secretion in collecting duct and increases net acid excretion [36,37,38]	Increased levels may help increase kidney acid excretion. Decreased levels may worsen acidosis [39,40]	Increased action associated with worsening kidney function. Blocking action slows progression [41,42]
Angiotensin II	Increased levels with metabolic acidosis [43]	Stimulates proximal tubule ammonia production and collecting duct acid excretion [44,45,46,47]	May help to preserve kidney acid excretion as kidney function declines [32]	A contributory cause of worsening kidney function. Blocking actions slows progression [32,42,48]
Endothelin	Increased with metabolic acidosis [49,50]	Increased net acid excretion by stimulating H^+^ secretion in proximal tubules and collecting duct [49,50,51,52,53]	May enhance acid excretion as kidney function declines [53,54]	Proinflammatory and profibrotic actions worsen kidney function [54,55,56]
PTH	Increased levels in some studies. [57,58]	Increased net acid excretion. Increased bone buffering capacity [17,58,59,60,61,62]	Increased levels in secondary hyperparathyroidism [63]	Association of secondary hyperparathyroidism with worsening CKD, but causal relationship is unclear [63,64]
Glucocorticoids	Increased levels [65]	Increased glutamine delivery and kidney ammonia production. increased proximal tubule acid secretion [66,67,68,69]	Higher levels of glucocorticoids in CKD are not associated with enhanced glutamine delivery. Unclear whether there is resistance to action [70]	Association of high glucocorticoids with progression but unclear whether the high glucocorticoid levels cause worsening of CKD [71,72]
Insulin	Increased levels due to insulin resistance [73,74]	High levels may reduce H^+^ secretion by distal nephron [75]	Effect on acid–base balance unclear. Correction of acidosis improves insulin sensitivity in CKD [76]	Hyperinsulinemia in the setting of insulin resistance may result in impaired kidney hemodynamics and progression of CKD [77,78,79]
Thyroid hormone	Reduced free T3 levels and increased TSH [80]	Hypothyroidism impedes maximal response to metabolic acidosis with reduced distal acidification and reduced tissue buffering [81,82,83,84,85]	Reduced T3 due to reduced conversion of T4. May improve with correction of acidosis [86]	Hypothyroidism in CKD is associated with poor outcomes (mortality, cardio-vascular disease, functional status, body composition) [87]
Antidiuretic hormone	Variable	May increase acid secretion in collecting duct leading to normal bicarbonate in SIADH [88,89]	Role in acid–base regulation in CKD is unclear as AVP signaling pathway may be disturbed [90]	Blocking action in patients with more rapidly progressive ADPKD can slow progression. [91,92]
Growth hormone (GH) and IGF-1	GH may be increased while IGF-1 levels may be decreased [93,94]	Reduced GH and IGF-1 activity may impair tissue buffering by loss of muscle and bone mass. Direct effects of GH on tubular acid secretion [93,95,96,97,98]	In severe CKD, GH levels are normal or increased but there is resistance to GH action accompanied by reduced IGF-1 levels. Unknown if GH receptors are reduced in renal tissue [98]	High GH levels may be associated with progressive kidney disease under certain conditions through adverse effects on podocytes [99,100,101,102]

## Data Availability

No original data were included in this review article.
